# Association between preoperative inflammatory biomarkers and postoperative pulmonary complications in stage I–II non-small cell lung cancer: a retrospective study

**DOI:** 10.3389/fmed.2026.1833302

**Published:** 2026-06-22

**Authors:** Jiao Liu, Shaowei Xin, Yang Yang, Qian Sun, Xiaodi Qi, Lei Hou, Changqi Ye

**Affiliations:** The 962nd Hospital of the Chinese People's Liberation Army Joint Logistics Support Force, Harbin, Heilongjiang, China

**Keywords:** inflammatory biomarkers, non-small cell lung cancer, propensity score matching, pulmonary complications, retrospective study

## Abstract

**Background:**

Postoperative pulmonary complications (PPCs) significantly impact outcomes in early-stage non-small cell lung cancer (NSCLC). The relationship between preoperative inflammatory biomarkers and PPCs in stage I–II NSCLC was assessed in this study.

**Methods:**

This retrospective analysis included 548 patients with stage I–II NSCLC who underwent surgery between 2016 and 2025. To control for confounding, 1:3 propensity score matching (PSM) was performed using clinical and surgical factors. Preoperative levels of the systemic immune-inflammation index (SII), neutrophil-to-lymphocyte ratio (NLR), platelet-to-lymphocyte ratio (PLR), and lymphocyte-to-monocyte ratio (LMR) were calculated. The associations with postoperative pulmonary complications (PPCs) were assessed using logistic regression. Discriminatory performance was evaluated using the area under the receiver operating characteristic curve (AUC), and comparisons were made with the DeLong test.

**Results:**

After PSM, 294 patients (75 with PPCs, 219 without) were analyzed, with well-balanced baseline characteristics. In multivariate analysis adjusted for BMI and surgical duration, elevated SII (adjusted OR for the highest quartile [Q4]: 5.53, 95% CI: 4.31–7.18, *p* < 0.001) and NLR (Q4 adjusted OR: 5.88, 95% CI: 3.27–9.18, *p* < 0.001) remained independently associated with PPCs. ROC curve analysis further showed that, among all inflammatory biomarkers, the SII was the best predictor of postoperative pulmonary complications, yet its AUC was merely 0.686 (95% CI: 0.617–0.755). In addition, DeLong’s test confirmed that SII had significantly superior discriminative ability compared to Single inflammatory indicators (all *p* < 0.05).

**Conclusion:**

Preoperative SII and NLR are independent predictors for PPCs in stage I–II NSCLC, with SII showing the best single-biomarker performance, albeit with a low AUC. Further research should explore combining SII with additional biomarkers to enhance predictive value.

## 1 Introduction

Non-small cell lung cancer (NSCLC) accounts for around 85% of all cases of lung cancer and is still the leading cause of cancer-related death globally (1). In 2022 alone, the disease caused over 1.8 million deaths, accounting for nearly 20% of total cancer mortality, with China bearing a disproportionate burden of more than 40% of both global incidence and deaths (2). For patients with early-stage (I–II) disease, surgical resection is the cornerstone of curative-intent therapy, with anatomic resections offering the optimal chance for long-term survival (3). The postoperative course, however, is frequently complicated by pulmonary complications including respiratory failure, acute respiratory distress syndrome, prolonged air leak, pneumonia, and atelectasis. These events carry substantial clinical significance, as they markedly increase short-term mortality, prolong hospitalization, elevate healthcare costs, and impair long-term functional recovery and quality of life (4). Furthermore, emerging evidence suggests that such complications may adversely affect oncological outcomes, potentially through delays in initiating adjuvant therapy and alterations to the perioperative immune environment (5, 6).

The pathophysiology of postoperative pulmonary complications (PPCs) is multifactorial, involving interactions between patient-related factors, surgical trauma magnitude, and the resultant systemic inflammatory response (7). Conventional risk stratification relies primarily on clinical parameters including age, smoking history, pulmonary function tests, with co-occurring conditions including chronic obstructive pulmonary disease (8–11). While useful, these models often fail to capture individual physiological reserve and subclinical vulnerability. Consequently, interest has grown in biological markers that more directly reflect the underlying systemic state, particularly those related to inflammation and immune response. Chronic systemic inflammation is a recognized hallmark of cancer progression and a key mediator in disease pathogenesis (12). In cancer surgery, a pre-existing pro-inflammatory state, exacerbated by surgical trauma, can induce a dysregulated immune milieu that predisposes to tissue damage, impaired healing, and increased infectious susceptibility (13).

Inflammatory biomarkers derived from routine complete blood count (CBC) differentials are particularly attractive for risk stratification due to their universal availability, low cost, and reproducibility (14). These composite indices integrate counts of different leukocyte subsets and platelets, potentially offering a more nuanced reflection of host inflammatory status and immune homeostasis than single parameters. The neutrophil-to-lymphocyte ratio (NLR) has been extensively studied across malignancies. It balances innate, pro-inflammatory neutrophil activity against adaptive, cell-mediated lymphocyte function (15). A preoperative NLR elevation suggests neutrophilia-driven inflammation with relative lymphocytopenia, indicating impaired immune surveillance (16). Prognostically, NLR is associated with poorer overall and recurrence-free survival in various cancers including NSCLC (17, 18), though its link to postoperative morbidity remains less defined and varies across surgical specialties. Similarly, the platelet-to-lymphocyte ratio (PLR) incorporates platelet count, reflecting platelets’ active roles in inflammatory and pro-thrombotic pathways (19). An elevated PLR may signify a hypercoagulable and pro-inflammatory state. In contrast, the equilibrium between lymphocytes and monocytes is represented by the lymphocyte-to-monocyte ratio (LMR). A lower LMR, indicating relative lymphopenia and monocytosis, is associated with immunosuppression and poorer oncological prognosis (20).

More recently, by simultaneously taking into consideration three crucial routes, the systemic immune-inflammation index (SII), which is computed as (platelets × neutrophils)/lymphocytes, has been suggested as a more thorough measure of systemic inflammation (21). The SII theoretically integrates information on inflammatory neutrophils, adaptive lymphocytes, and pro-thrombotic platelets, and has demonstrated superior prognostic value for survival in several solid tumors compared to other indices (22, 23). Its utility in predicting non-oncological surgical outcomes, specifically PPCs in lung cancer surgery, remains an underexplored area of investigation.

The current evidence for these inflammatory biomarkers in thoracic surgery remains fragmented and inconsistent. Many studies prioritize their long-term prognostic value, whereas postoperative complications are frequently analyzed as secondary endpoints or aggregated with cardiac or infectious events in composite measures—an approach that obscures the specific relationship between systemic inflammation and pulmonary morbidity. Moreover, investigations often include heterogeneous cohorts with advanced disease, in whom neoadjuvant therapy may substantially alter systemic inflammatory profiles and confound the interpretation of preoperative biomarkers.

This retrospective study seeks to rigorously evaluate the association between preoperative inflammatory biomarkers and clinically significant PPCs. Establishing a strong, independent association would support the integration of these readily accessible biomarkers into existing preoperative risk-assessment frameworks. Such integration could facilitate more personalized patient counseling and tailored perioperative strategies, ultimately aiming to mitigate postoperative risk and enhance this population’s clinical results.

## 2 Methods

### 2.1 Study design

**Figure 1:**
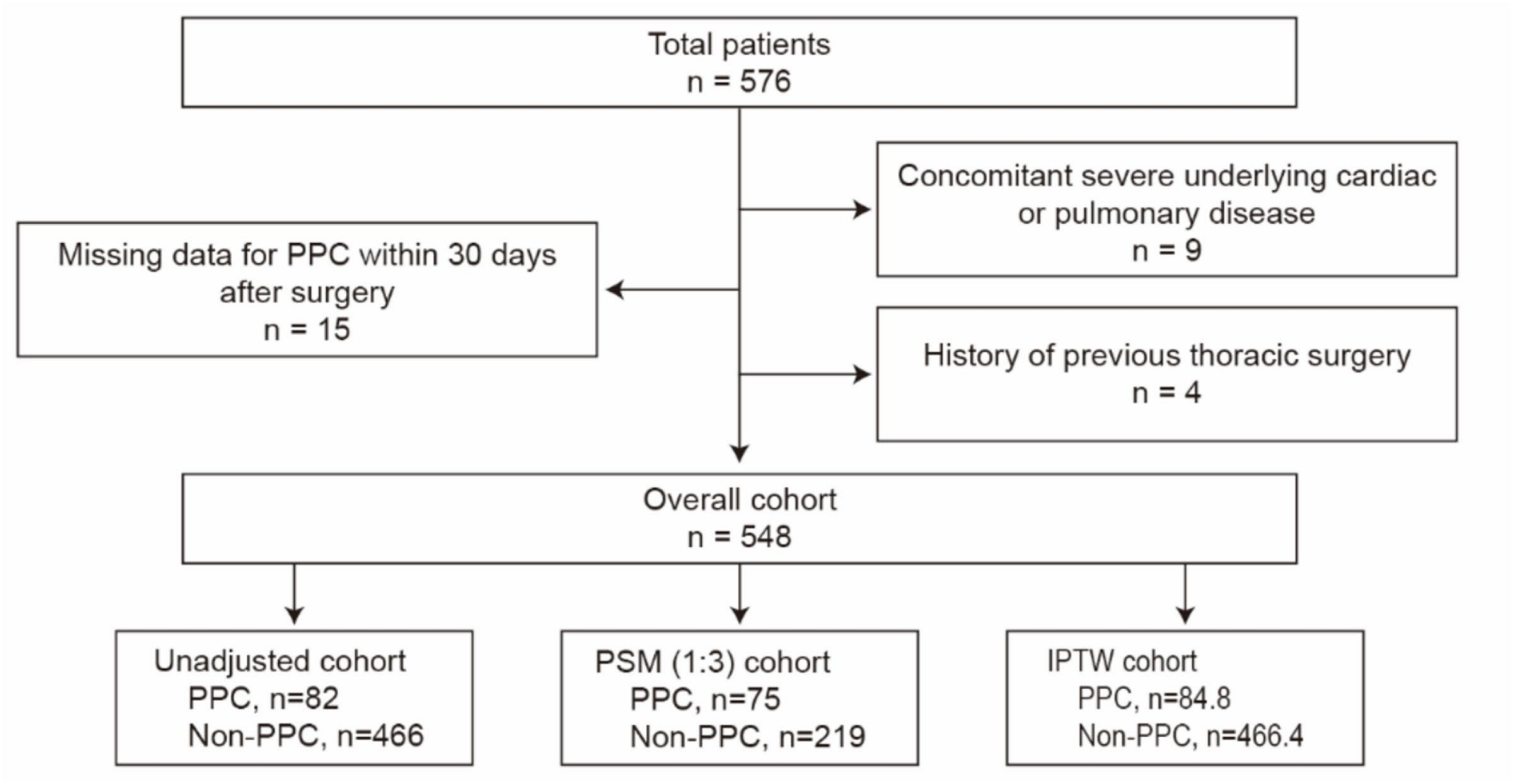
Flowchart of patient selection. PPC, postoperative pulmonary complication; PSM, propensity-score matching; IPTW, inverse probability of treatment weighting.

A retrospective analysis was carried out. Patients diagnosed with stage I–II NSCLC who underwent surgical treatment at our institution between January 2016 and December 2025 were screened for eligibility. Inclusion criteria: (a) Pathologically confirmed stage I–II NSCLC, in accordance with the diagnostic standards of the NCCN Clinical Practice Guidelines in Oncology for NSCLC (24); (b) Karnofsky Performance Status (KPS) score of ≥ 60 (25); (c) Underwent anatomical lung resection (lobectomy, segmentectomy, or pneumonectomy); and (d) Accessibility of comprehensive laboratory and clinical data. Excluded criteria: (a) Severe underlying cardiac or pulmonary disease; (b) History of previous thoracic surgery; (c) Active infection or inflammatory diseases; (d) Previous chemotherapy or radiotherapy. Finally, a total of 548 patients were included in the analysis (Figure 1). All patients received standardized perioperative care, which encompassed prophylactic antibiotics, chest physiotherapy, and consistent postoperative monitoring.

This study was conducted in accordance with the ethical principles of the Declaration of Helsinki. The study protocol was reviewed and approved by the Institutional Ethics Committee of the 962nd Hospital of the Chinese People’s Liberation Army Joint Logistics Support Force (No. 202620). Because the study was retrospective in nature, informed permission was not required.

### 2.2 Data collection

A comprehensive set of perioperative data was systematically collected, including: (a) Demographics and Clinical Characteristics: age, sex, body mass index (BMI), smoking history, alcohol consumption history, and preoperative comorbidities; (b) Tumor and Pathological Features: final pathological TNM (Tumor, Node, Metastasis) stage; (c) Surgical Details: surgical approach (uniportal VATS, multiportal VATS, or thoracotomy), type of lung resection (lobectomy, segmentectomy, pneumonectomy), and surgical duration.

### 2.3 Preoperative inflammatory biomarkers

Preoperative inflammatory indices were derived from CBC differentials obtained within 1 week before surgery. The following biomarkers were computed using absolute numbers of neutrophils, lymphocytes, monocytes, and platelets (×10^9^/L) and the following formulas: NLR, neutrophil count/lymphocyte count; SII, platelet count × neutrophil count/lymphocyte count; LMR, lymphocyte count/monocyte count; PLR, platelet count/lymphocyte count.

### 2.4 Postoperative pulmonary complications (PPCs)

Patients were stratified based on the occurrence of any pulmonary complication within 30 days postoperatively. Pulmonary complications included atelectasis requiring bronchoscopy, pneumonia, pleural effusion necessitating drainage, chylothorax, pneumothorax, prolonged air leak (>5 days), or respiratory failure. To standardise the assessment of complication severity, all events were graded according to the Clavien-Dindo classification system (26–28). A clinically significant postoperative complication was defined as any such event graded as Class II or higher. This classification system has been validated in diverse surgical populations, offers high reproducibility, and has been widely adopted in studies of postoperative outcomes after lung cancer surgery across different countries (8, 29, 30). Therefore, it provides a reliable and established framework for defining the primary outcome in the present study.

### 2.5 Statistical analysis

Continuous variables with a normal distribution were compared using Student’s *t*-test and are presented as mean ± standard deviation. For continuous variables that were not normally distributed, the Wilcoxon rank-sum test was employed, and the findings were presented as the median and interquartile range (25th–75th percentiles). The Chi-square test or Fisher’s exact test were used to compare categorical variables, and the results are shown as percentages and frequencies.

To reduce the selection bias, a 1:3 propensity score matching (PSM) and inverse probability of treatment weighting (IPTW) were carried out utilizing the nearest-neighbor matching technique (Figure 1). To avoid inadequate matching, a caliper radius of 0.1 standard deviation was established. A logistic regression model using the following baseline factors was used to estimate the propensity score: Age, BMI, surgical duration, sex, smoking history, alcohol consumption history, comorbidities, TNM stage, type of lung resection, and surgical approach. Standardized mean differences (SMD) were used to evaluate covariate balance following matching; SMD < 0.10 indicated good balance (31). In the PSM cohort, univariable logistic regression was first applied to evaluate the association between preoperative inflammatory indices and the incidence of PPCs. Subsequently, two multivariable logistic models were constructed: Model 2 adjusted for covariates with SMD > 0.1, and Model 3 further adjusted for clinically important covariates based on Model 2. IPTW was employed as a sensitivity analysis to verify the robustness of the results. The area under the curve (AUC) for the preoperative inflammatory biomarkers was calculated using receiver operating characteristic (ROC) curve analysis. The DeLong test was subsequently applied to compare the discriminatory performance between single inflammatory indicators and composite inflammatory indices in predicting PPCs risk.

All statistical tests were two-sided, and a *p*-value of less than 0.05 was considered statistically significant. For every analysis, R 4.5.1 and SPSS 26.0 were utilized.

## 3 Results

### 3.1 Patient characteristics

PSM (1:3 ratio) and IPTW were applied to balance baseline covariates. After PSM, the cohort sizes were reduced from 82 to 75 in the PPC group and from 466 to 219 in the non-PPC group. In the IPTW cohort, effective sample sizes were 84.8 and 466.4, respectively. All baseline variables showed no significant differences between groups in the PSM cohort (all *p* > 0.05) and the IPTW cohort (all *p* > 0.05), with SMD values below 0.1 for most variables. Minor residual imbalances were observed for BMI (SMD = 0.201) and surgical duration (SMD = 0.221) in the PSM cohort (Table 1).

**Table 1:** Baseline characteristics of patients.

Variables	Unadjusted cohort				PSM cohort				IPTW cohort			
	PPC (*n* = 82)	Non-PPC (*n* = 466)	*p*	SMD	PPC (*n* = 75)	Non-PPC (*n* = 219)	*p*	SMD	PPC (*n* = 84.8)	Non-PPC (*n* = 466.4)	*p*	SMD
Age (years)	68.5 ± 7.2	65.1 ± 8.5	0.001	0.409	67.6 ± 6.9	67.3 ± 7.7	0.821	0.042	67.4 ± 7.1	67.3 ± 7.3	0.812	0.024
BMI (kg/m^2^)	23.8 ± 3.5	24.2 ± 3.3	0.316	0.120	24.3 ± 2.8	23.7 ± 3.1	0.130	0.201	23.9 ± 3.0	24.0 ± 3.1	0.587	0.110
Surgical duration (min)	182.1 ± 45.4	165.2 ± 38.2	<0.001	0.429	183.3 ± 34.7	175.2 ± 39.3	0.118	0.221	180.5 ± 37.8	176.2 ± 38.1	0.061	0.318
Sex			0.886	0.025			0.845	0.033			0.873	0.028
Male	52 (63.4)	301 (64.6)			47 (62.7)	140 (63.9)			53.8 (63.5)	302.2 (64.8)		
Female	30 (36.6)	165 (35.4)			28 (37.3)	79 (36.1)			31.0 (36.5)	164.2 (35.2)		
Smoking history	58 (70.7)	280 (60.1)	0.067	0.225	52 (69.3)	151 (68.9)	0.951	0.011	52.2 (61.5)	287.8 (61.7)	0.941	0.010
Alcohol consumption history	25 (30.5)	140 (30.0)	0.937	0.010	22 (29.3)	67 (30.6)	0.838	0.034	25.4 (30.0)	140.6 (30.1)	0.827	0.032
Comorbidities	55 (67.1)	252 (54.1)	0.029	0.268	50 (66.7)	145 (66.2)	0.942	0.014	56.3 (66.4)	310.1 (66.5)	0.935	0.013
TNM stage			0.022	0.258			0.781	0.043			0.765	0.040
IA or IB	60 (73.2)	390 (83.7)			56 (74.7)	167 (76.3)			63.5 (74.9)	349.8 (75.0)		
IIA or IIB	22 (26.8)	76 (16.3)			19 (25.3)	52 (23.7)			21.3 (25.1)	116.6 (25.0)		
Type of lung resection			0.041	0.220			0.905	0.027			0.892	0.025
Lobectomy	70 (85.4)	430 (92.3)			66 (88.0)	194 (88.6)			75.2 (88.7)	414.2 (88.8)		
Segmentectomy	8 (9.8)	30 (6.4)			7 (9.3)	21 (9.6)			7.7 (9.1)	42.3 (9.1)		
Pneumonectomy	4 (4.9)	6 (1.3)			2 (2.7)	4 (1.8)			1.9 (2.2)	9.9 (2.1)		
Surgical approach			0.093	0.229			0.882	0.053			0.860	0.048
U-VATS	40 (48.8)	280 (60.1)			38 (50.7%)	117 (53.4%)			44.1 (52.0)	242.7 (52.0)		
M-VATS	35 (42.7)	165 (35.4)			32 (42.7%)	90 (41.1%)			35.7 (42.1)	197.1 (42.3)		
Thoracotomy	7 (8.5)	21 (4.5)			5 (6.6%)	12 (5.5%)			5.0 (5.9)	26.6 (5.7)		

PPC, postoperative pulmonary complication; BMI, body mass index; TNM, Tumor, Node, Metastasis; U-VATS, uniportal video-assisted thoracic surgery; M-VATS, multiportal video-assisted thoracic surgery; SMD, standardized mean difference.

### 3.2 Patient preoperative inflammatory biomarkers

As shown in Table 2, in the PSM cohort, PPC patients exhibited higher neutrophil counts (5.21 ± 1.63 vs. 4.52 ± 1.50 × 10^9^/L, *p* = 0.001) and lower lymphocyte counts (1.55 ± 0.41 vs. 1.79 ± 0.53 × 10^9^/L, *p* = 0.001). Consequently, the SII (907.23 ± 417.91 vs. 651.60 ± 319.41, *p* < 0.001), NLR (3.60 ± 1.51 vs. 2.78 ± 1.26, *p* < 0.001), and PLR (174.10 ± 52.72 vs. 144.91 ± 57.43, *p* < 0.001) were significantly higher, while LMR was lower (3.43 ± 1.96 vs. 4.02 ± 2.08, *p* = 0.034) in the PPC group. These findings were largely confirmed by IPTW analysis: platelets (249.81 ± 52.32 vs. 242.18 ± 53.62 × 10^9^/L, *p* = 0.018), neutrophils (4.85 ± 1.58 vs. 4.61 ± 1.55 × 10^9^/L, *p* = 0.012), and lymphocytes (1.63 ± 0.48 vs. 1.74 ± 0.51 × 10^9^/L, *p* = 0.007) remained significantly different, as did SII (845.62 ± 398.71 vs. 730.24 ± 362.47, *p* < 0.001), NLR (3.35 ± 1.48 vs. 2.95 ± 1.41, *p* < 0.001), and PLR (168.51 ± 54.94 vs. 151.32 ± 55.21, *p* < 0.001). However, the difference in LMR did not reach statistical significance in the IPTW cohort (3.65 ± 2.01 vs. 3.88 ± 2.06, *p* = 0.062).

**Table 2:** Preoperative inflammatory biomarkers of patients.

Variables	Unadjusted cohort			PSM cohort			IPTW cohort		
	PPC (*n* = 82)	Non-PPC (*n* = 466)	*p*	PPC (*n* = 75)	Non-PPC (*n* = 219)	*p*	PPC (*n* = 84.8)	Non-PPC (*n* = 466.4)	*p*
Single inflammatory indicators									
Platelets (×10^9^ /L)	255.36 ± 58.41	223.15 ± 51.73	<0.001	254.46 ± 48.98	236.56 ± 59.38	0.019	249.81 ± 52.32	242.18 ± 53.62	0.018
Neutrophils (×10^9^ /L)	5.21 ± 1.87	4.05 ± 1.52	<0.001	5.21 ± 1.63	4.52 ± 1.50	0.001	4.85 ± 1.58	4.61 ± 1.55	0.012
Lymphocytes (×10^9^ /L)	1.45 ± 0.51	1.82 ± 0.63	<0.001	1.55 ± 0.41	1.79 ± 0.53	0.001	1.63 ± 0.48	1.74 ± 0.51	0.007
Monocytes (×10^9^ /L)	0.58 ± 0.21	0.52 ± 0.19	0.010	0.52 ± 0.15	0.50 ± 0.15	0.317	0.52 ± 0.16	0.51 ± 0.15	0.396
Composite inflammatory indices									
SII	980.50 ± 502.36	505.30 ± 385.47	<0.001	907.23 ± 417.91	651.60 ± 319.41	<0.001	845.62 ± 398.71	730.24 ± 362.47	<0.001
PLR	185.74 ± 55.62	128.45 ± 50.33	<0.001	174.10 ± 52.72	144.91 ± 57.43	<0.001	168.51 ± 54.94	151.32 ± 55.21	<0.001
NLR	3.78 ± 1.85	2.35 ± 1.24	<0.001	3.60 ± 1.51	2.78 ± 1.26	<0.001	3.35 ± 1.48	2.95 ± 1.41	<0.001
LMR	2.55 ± 1.12	3.55 ± 1.48	<0.001	3.43 ± 1.96	4.02 ± 2.08	0.034	3.65 ± 2.01	3.88 ± 2.06	0.062

PPC, postoperative pulmonary complication; SII, systemic immune-inflammation index; NLR, neutrophil-to-lymphocyte ratio; PLR, platelet-to-lymphocyte ratio; LMR, lymphocyte-to-monocyte ratio; SMD, standardized mean difference.

### 3.3 Association between inflammatory biomarkers and PPCs risk

**Figure 2:**
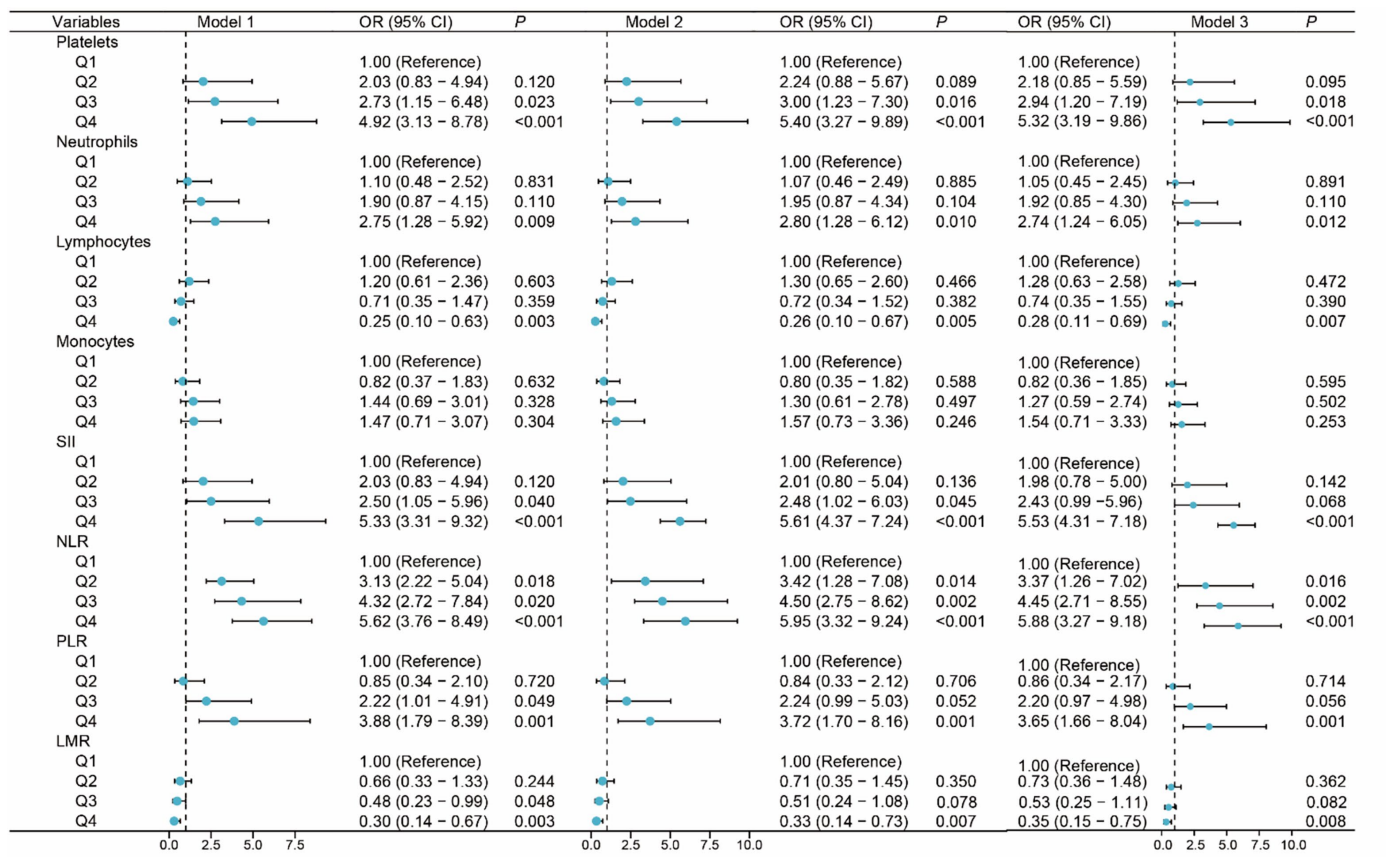
Forest plots display the risk association between preoperative inflammatory biomarkers and PPCs. Model 1: Unadjusted for covariates; Model 2: Adjusted for BMI and surgical duration; Model 3: Adjusted for age, comorbidities, TNM stage, and type of lung resection based on Model 2. BMI, body mass index; TNM, Tumor, Node, Metastasis; PPC, postoperative pulmonary complication; SII, systemic immune-inflammation index; NLR, neutrophil-to-lymphocyte ratio; PLR, platelet-to-lymphocyte ratio; LMR, lymphocyte-to-monocyte ratio.

Figure 2 presents the association between preoperative inflammatory biomarkers and the risk of PPCs in the matched cohort. In the unadjusted analysis (Model 1), elevated levels of several biomarkers were significantly associated with an increased risk of PPCs. The highest quartile (Q4) of the SII (OR: 5.33, 95% CI: 3.31–9.32, *p* < 0.001) and NLR (OR: 5.62, 95% CI: 3.76–8.49, *p* < 0.001) demonstrated the strongest associations. Platelet counts (Q4 OR: 4.92, 95% CI: 3.13–8.78, *p* < 0.001), neutrophil counts (Q4 OR: 2.75, 95% CI: 1.28–5.92, *p* = 0.009), and lymphocyte counts (Q4 OR: 0.25, 95% CI: 0.10–0.63, *p* = 0.003) were also significant predictors. Model 3 adjustment for BMI, surgical duration, age, comorbidities, TNM stage, and type of lung resection, the associations for the highest quartiles of SII (adjusted OR: 5.53, 95% CI: 4.31–7.18, *p* < 0.001) and NLR (adjusted OR: 5.88, 95% CI: 3.27–9.18, *p* < 0.001) remained robust and statistically significant.

**Figure 3:**
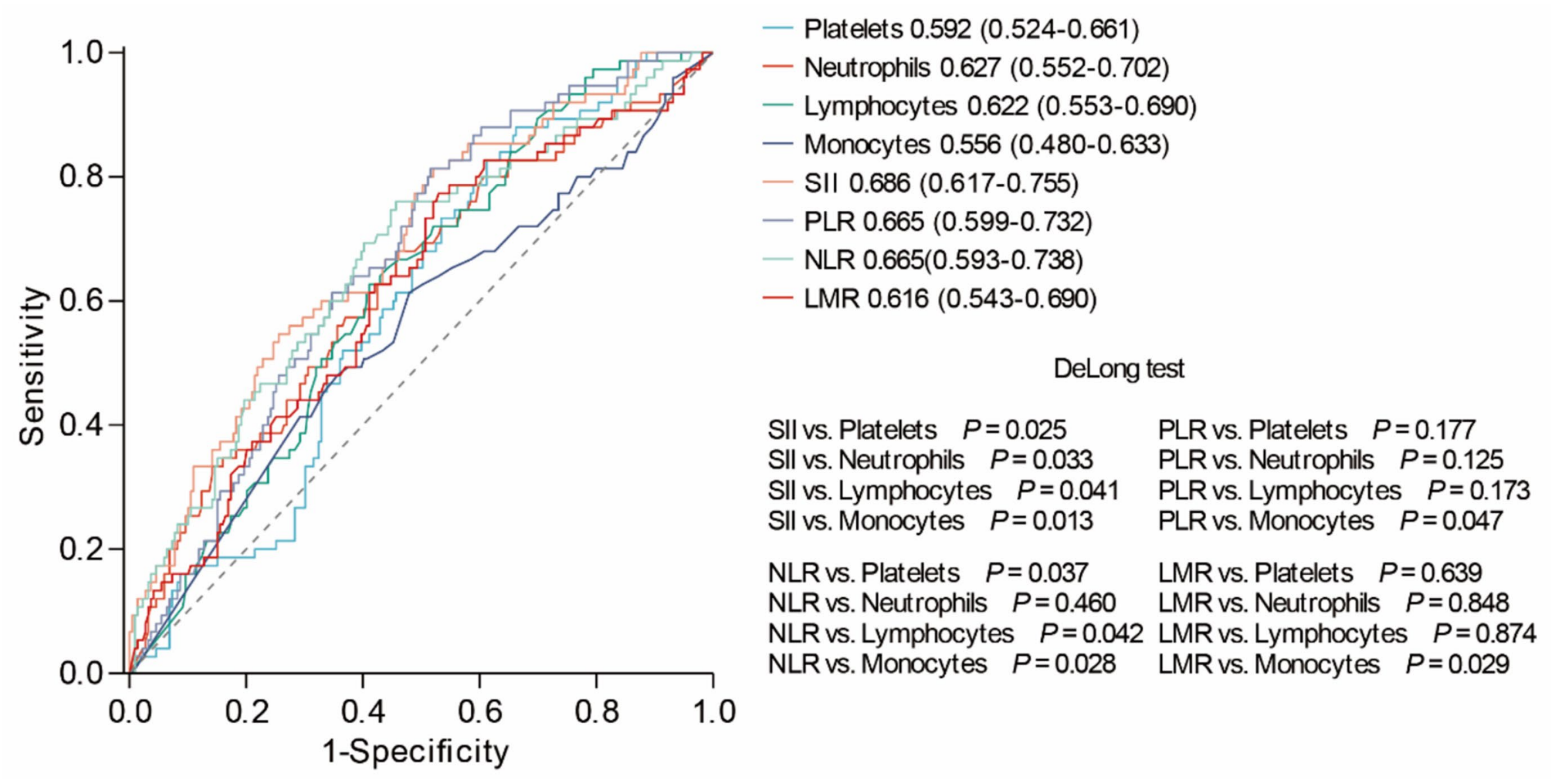
ROC curve evaluation of the predictive performance of preoperative inflammatory biomarkers for PPCs risk. ROC, Receiver operating characteristic; PPCs, postoperative pulmonary complication; SII, systemic immune-inflammation index; NLR, neutrophil-to-lymphocyte ratio; PLR, platelet-to-lymphocyte ratio; LMR, lymphocyte-to-monocyte ratio.

The discriminative ability of individual and combined inflammatory biomarkers for PPCs was assessed using ROC curve analysis (Figure 3). Among all indicators, the SII demonstrated the highest AUC of 0.686 (95% CI: 0.617–0.755). The NLR and PLR both yielded an AUC of 0.665 (95% CIs: 0.593–0.738 and 0.599–0.732, respectively). The LMR and individual cell counts showed lower predictive values, with AUCs ranging from 0.556 to 0.627. DeLong’s test further revealed that SII possessed significantly superior discriminative ability over platelets, neutrophils, lymphocytes, and monocytes (all *p* < 0.05), and that NLR also exhibited significantly superior discriminative ability over platelets, lymphocytes, and Monocytes (all *p* < 0.05). (Figure 3).

## 4 Discussion

This study shows that in individuals undergoing curative-intent surgery for stage I–II NSCLC, preoperative systemic inflammation is independently linked to a higher incidence of PPCs. Following rigorous adjustment for confounders via PSM and multivariate regression, the SII and NLR emerged as the strongest predictors. SII exhibited the highest discriminative ability. These findings underscore the critical role of the preoperative inflammatory state in determining surgical outcomes and propose a practical adjunct to conventional risk stratification models.

The superior predictive performance of SII compared to other indices, including NLR, is supported by existing literature and can be explained by its more comprehensive pathophysiological integration (32–34). Although NLR is a well-validated prognostic marker in NSCLC, it is limited to reflecting the balance between neutrophils and lymphocytes. Our results are consistent with prior studies in gastrointestinal and hepatobiliary malignancies where SII showed superior prognostic value, indicating its broader applicability (35). A key advantage of SII is its incorporation of platelet count, which represents a third critical pathway. Platelets are active contributors to systemic inflammation and cancer-associated thrombosis, releasing cytokines and growth factors that can exacerbate tissue injury and impede healing (36). Consequently, SII simultaneously accounts for neutrophilic inflammation, relative lymphocytopenia, and thrombocytosis, offering a more holistic assessment of the pro-inflammatory, pro-thrombotic, and immunosuppressive milieu associated with increased surgical risk.

The independent association between elevated inflammatory biomarkers and PPCs, which persists after adjustment for operative and physiological confounders, suggests that their predictive value extends beyond serving as simple proxies for frailty or surgical stress. In contrast to some previous studies where such associations were attenuated after multivariate adjustment, our findings indicate that these biomarkers reflect a specific, intrinsic biological vulnerability (37, 38). This vulnerability is rooted in chronic, cancer-associated inflammation, a state sustained by tumor-derived signals and the host immune response (39). Within this context, major surgery acts as a potent immunological stressor. It amplifies the pre-existing inflammatory cascade and can trigger a dysregulated systemic immune response, which clinically manifests as infectious or inflammatory pulmonary complications (40). Our study focused on a homogeneous cohort of treatment-naïve stage I–II NSCLC patients, strengthens this interpretation by minimizing the substantial confounding effects of neoadjuvant therapy on hematological parameters, thereby providing a clearer view of baseline inflammation as a genuine risk factor.

From a translational perspective, the practical utility of SII, NLR, and similar indices is a major strength. Derived from routine, low-cost, and universally available preoperative blood tests, they offer an easily accessible means of risk assessment without requiring specialized equipment. This facilitates their potential integration into existing preoperative protocols across diverse healthcare settings. Patients identified as high-risk based on an elevated SII could be candidates for enhanced perioperative care pathways. Such pathways might include prehabilitation programs focused on nutrition and physical conditioning, meticulous intraoperative management employing lung-protective strategies, and intensified postoperative monitoring and physiotherapy (41, 42). However, the relatively modest AUC values, all below 0.70, necessitate caution. These values indicate that while the biomarkers provide valuable incremental information, their standalone diagnostic accuracy is insufficient for use as sole screening tools. Their optimal application lies within multifactorial risk models, where they complement rather than replace traditional assessments such as pulmonary function tests and performance status evaluation. Future research should investigate whether combining SII with a fundamentally different type of biomarker could yield a more synergistic improvement.

Within the context of existing literature, our findings corroborate and extend previous work. Numerous studies have established links between NLR, and more recently SII, and poorer long-term oncological outcomes across various cancers, including NSCLC (22, 43, 44). However, research specifically focusing on their role in predicting postoperative complications, particularly pulmonary morbidity in early-stage lung cancer, has been less consistent. This inconsistency often stems from heterogeneous study populations and the use of composite endpoints. Our study strengthens the evidence by demonstrating this association in a well-defined, propensity score-matched cohort using a specific pulmonary complication endpoint. Furthermore, it adds nuance by directly comparing the performance of multiple indices and demonstrating the lack of additive value from a combined model of raw cell counts, a practical insight frequently absent from prior studies.

Several limitations of this study must be acknowledged. First, despite our use of propensity score correction, selection bias and unmeasured confounding are intrinsic concerns of the retrospective, single-center approach. Second, although we employed a standardized classification system, the retrospective identification of PPCs may be subject to variability in clinical documentation. Third, our results may only be applicable in comparable clinical circumstances, which emphasizes the necessity of external validation in prospective, multicenter cohorts. Fourth, our reliance on a single preoperative blood measurement precludes any assessment of dynamic perioperative changes in inflammatory markers, which might offer additional prognostic information. Finally, the observed associations do not establish causality; future interventional studies are necessary to determine whether targeted modulation of preoperative inflammation can effectively reduce the risk of PPCs.

## 5 Conclusion

In conclusion, this study identifies preoperative SII and NLR as independent and practical biomarkers for predicting pulmonary complications after surgery for early-stage NSCLC. SII, with its composite nature, shows the most promise as a single indicator. These findings advocate for the integration of systemic inflammation assessment into routine preoperative risk profiling. Future prospective research should focus on validating these indices in broader settings, exploring their dynamic perioperative changes, and, importantly, evaluating their combination with other clinical or laboratory markers to enhance predictive accuracy, as well as testing whether targeted interventions for patients with high inflammatory burden can improve surgical outcomes.

## Data Availability

The original contributions presented in the study are included in the article/supplementary material, further inquiries can be directed to the corresponding author.
